# Evaluation of an oxygen‐dependent self‐inducible surfactin synthesis in *B. subtilis* by substitution of native promoter P_*srfA*_ by anaerobically active P_*narG*_ and P_*nasD*_

**DOI:** 10.1186/s13568-021-01218-4

**Published:** 2021-04-19

**Authors:** Mareen Hoffmann, Alina Braig, Diana Stephanie Fernandez Cano Luna, Katharina Rief, Philipp Becker, Chantal Treinen, Peter Klausmann, Kambiz Morabbi Heravi, Marius Henkel, Lars Lilge, Rudolf Hausmann

**Affiliations:** Institute of Food Science and Biotechnology (150), Department of Bioprocess Engineering (150k), University of Hohenheim, Fruwirthstr. 12, 70599 Stuttgart, Germany

**Keywords:** *Bacillus subtilis*, Lipopeptide biosurfactants, Surfactin, Microaerobic, Promoter exchange, Oxygen

## Abstract

A novel approach targeting self-inducible surfactin synthesis under oxygen-limited conditions is presented. Because both the nitrate (NarGHI) and nitrite (NasDE) reductase are highly expressed during anaerobic growth of *B. subtilis*, the native promoter P_*srfA*_ of the surfactin operon in strain *B. subtilis* JABs24 was replaced by promoters P_*narG*_ and P_*nasD*_ to induce surfactin synthesis anaerobically. Shake flask cultivations with varying oxygen availabilities indicated no significant differences in native P_*srfA*_ expression. As hypothesized, activity of P_*narG*_ and P_*nasD*_ increased with lower oxygen levels and surfactin was not produced by P_*srfA*_::P_*narG*_ as well as P_*srfA*_::P_*nasD*_ mutant strains under conditions with highest oxygen availability. P_*narG*_ showed expressions similar to P_*srfA*_ at lowest oxygen availability, while maximum value of P_*nasD*_ was more than 5.5-fold higher. Although the promoter exchange P_*srfA*_::P_*narG*_ resulted in a decreased surfactin titer at lowest oxygen availability, the strain carrying P_*srfA*_::P_*nasD*_ reached a 1.4-fold increased surfactin concentration with 696 mg/L and revealed an exceptional high overall *Y*_P/X_ of 1.007 g/g. This value also surpassed the *Y*_P/X_ of the reference strain JABs24 at highest and moderate oxygen availability. Bioreactor cultivations illustrated that significant cell lysis occurred when the process of “anaerobization” was performed too fast. However, processes with a constantly low agitation and aeration rate showed promising potential for process improvement, especially by employing the strain carrying P_*srfA*_::P_*nasD*_ promoter exchange. Additionally, replacement of other native promoters by nitrite reductase promoter P_*nasD*_ represents a promising tool for anaerobic-inducible bioprocesses in *Bacillus*.

**Supplementary Information:**

The online version contains supplementary material available at 10.1186/s13568-021-01218-4.

## Introduction

The cyclic lipopeptide surfactin synthesized by *Bacillus subtilis* displays promising characteristics in a variety of industrial sectors (Geissler et al. 2019a) due to its excellent surface-active properties and antimicrobial activities (Falardeau et al. 2013; Li et al. 2019). In addition, surfactin is a promising alternative to surfactants of petrochemical and oleochemical origin (Henkel et al. 2017). However, two bottlenecks regarding surfactin production must be mentioned that are often addressed in research. First, the excessive foaming during conventional aerobic processes, and second, the overall low titers of wild-type strains that are insufficient for industrial production.

Studies reported both on difficulties in operation due to uncontrolled foaming and on a great loss of cultivation medium at high agitation and aeration rates (Davis et al. 2001; Yao et al. 2015; Yeh et al. 2006). If product separation by foaming is integrated into the process as a first surfactin enrichment, cells are also lost for further surfactin production (Willenbacher et al. 2014). Issues with blocked exhaust air filters and hence increase in pressure can also occur. However, high agitation and aeration rates are indispensable when defined oxygen levels shall be maintained in commonly employed aerobic cultivations. Indeed, Yeh et al. (2006) reported that up to a certain high level of aeration and stirrer speed surfactin synthesis was improved in carrier-assisted cultivation due to improved oxygen transfer rate and mass transfer efficiency. In contrast to these circumstances, other studies described that an enhanced surfactin production rate was reached in oxygen-limited conditions (Davis et al. 1999; Kim et al. 1997). In this sense, the set-points for both aeration and agitation rate are crucial to have the optimal performance in a bioreactor cultivation with a defined strain. Interestingly, an anaerobic cultivation of *B. subtilis* as demonstrated by Willenbacher et al. (2015) resulted in a completely foam-free approach for surfactin formation. Nevertheless, anaerobic nitrate respiration reveals some restrictions. Both the overall low growth rates of *B. subtilis* producer strains and comparatively low surfactin titers make these processes inferior to aerobic counterparts. Still, promising and high values with regard to product per biomass yields were obtained (Geissler et al. 2019b; Willenbacher et al. 2015). Another aspect is the negative impact of nitrite as well as acetate on anaerobic cell growth. Especially, the latter one increased drastically throughout nitrate respiration of *B. subtilis* and as such is an interesting candidate for strain engineering (Hoffmann et al. 2020).

With respect to the overall low surfactin titers of wild-type strains, rational strain engineering is also often employed, as heterologous production of surfactin in other host strains is difficult to realize (Hu et al. 2019). In this field, substitution of the native promoter P_*srfA*_, whose regulation is dependent on a complex quorum-sensing mechanism (Geissler et al. 2019a), constitutes one promising approach. The replacement of P_*srfA*_ by constitutively promoters was reported in several studies (Coutte et al. 2010a; Willenbacher et al. 2016). Results demonstrated that both gene expression during the time course of cultivation as well as the ability of the wild-type strain to produce surfactin influence final surfactin concentrations. In contrast to constitutive promoters, several studies reported on improved surfactin titers up to 17-fold employing IPTG-inducible promoters (Jiao et al. 2017; Sun et al. 2009; Wang et al. 2018). However, IPTG is rather expensive, which poses a bottleneck for large-scale production.

The current study aimed to address the afore mentioned aspects and examined an oxygen-dependent, self-inducible expression system employing the promoters of the nitrate reductase P_*narG*_ and the nitrite reductase P_*nasD*_. Hence, the presented method takes advantage of the ability of *B. subtilis* to grow anaerobically by nitrate respiration, the most effective life style to generate ATP after aerobic growth with oxygen as electron acceptor (Härtig and Jahn 2012). The adaptation of aerobic growing cells to anaerobic conditions is dependent on the interplay of three major regulators, which are ResDE, Fnr and Rex (a detailed overview is given by Härtig and Jahn (2012)). In a first step, nitrate is reduced to nitrite by the catabolic nitrate reductase NarGHI (Nakano et al. 1998). In a second step, nitrite is further reduced to ammonium by the nitrite reductase NasDE. Indeed, NasDE is also involved in aerobic assimilatory reduction of nitrate to nitrite. However, the expression of NasDE was reported to be significantly induced in the absence of oxygen emanating from a promoter located in between *nasC* and *nasD* (Nakano et al. 1998). Hence, both P_*narG*_ and P_*nasD*_ are involved in anaerobic nitrate respiration, although also the availability of nitrogen sources plays an important role in this issue. In addition to these anaerobically inducible promoters, *B. subtilis* exhibits more regulatory networks that allow induction of gene expression under oxygen limitation. An overview of *B. subtilis* transcriptome under anaerobic growth was provided by Nicolas et al. (2012). Hence, further promising promoters for anaerobic induction of gene expression are P_*lctE*_, P_*alsS*_ and P_*hmp*_.

We hypothesized that the presented self-inducible system poses an interesting novel cultivation strategy to synthesize surfactin under oxygen-limited conditions resulting in reduced foam formation. The proposed system is not only promising for foaming agents like surfactin, but also for other oxygen-sensitive target products. On top, preliminary investigations in bioreactor cultivations to establish a simple and robust cultivation strategy using a native *B. subtilis* surfactin producer strain and derivative strains with promoter exchanges are presented. Emerging limitations of bioreactor cultivations will be emphasized that display a promising starting position for further optimizations.

## Materials and methods

### Chemicals and materials

All chemicals (Carl Roth GmbH & Co. KG, Karlsruhe, Germany) were of analytical grade. The reference standard for surfactin (≥ 98% purity) and glucose were from Sigma-Aldrich Laborchemikalien GmbH (Seelze, Germany).

### Microorganisms and genetic engineering

All strains are listed in Table 1 including strain *B. subtilis* JABs24 (Geissler et al. 2019b) and derivatives thereof. Chemical competent *E. coli* BL21-Gold strains were used for plasmid propagation. Plasmids and primers (Eurofins Genomics GmbH, Ebersberg, Germany) used for strain construction are summarized in Additional file 1: Table S1 and Table S2. Transformants were selected on LB agar plates supplemented with either ampicillin (100 µg/mL), spectinomycin (125 µg/mL) or erythromycin (10 µg/mL for *E. coli*, 5 µg/mL for *B. subtilis*).

**Table 1:** Overview of strains used in the current study

Strain	Genotype or description	References
*B. subtilis*		
JABs24	*trp*^+^ *sfp*^+^ Δ*manPA*	Geissler et al. (2019b)
MG1	*trp*^+^ *sfp*^+^ Δ*manPA amyE*::[P_*narG*_-*lacZ*, *spcR*]	Hoffmann et al. (2020)
MG5	*trp*^+^ *sfp*^+^ Δ*manPA amyE*::[P_*nasD*_-*lacZ*, *spcR*]	Hoffmann et al. (2020)
KM1016	*trp*^+^ *sfp*^+^ Δ*manPA amyE*::[P_*srfA*_-*lacZ*, *spcR*]	This study
MG11	*trp*^+^ *sfp*^+^ Δ*manPA* P_*srfA*_::P_*narG*_	This study
MG12	*trp*^+^ *sfp*^+^ Δ*manPA* P_*srfA*_::P_*narG*_ *amyE*::[P_*narG*_-*lacZ*, *spcR*]	This study
MG13	*trp*^+^ *sfp*^+^ Δ*manPA* P_*srfA*_::P_*nasD*_	This study
MG14	*trp*^+^ *sfp*^+^ Δ*manPA* P_*srfA*_::P_*nasD*_ *amyE*::[P_*nasD*_-*lacZ*, *spcR*]	This study
*E. coli*		
BL21-Gold (DE3)	*F*^*-*^ *ompT hsdS(rB*^*-*^ *mB*^*-*^*) dcm*^*+*^ Tet^*r*^ *gal λ*(DE3) *endA* Hte	Agilent, Waldbronn, Germany

For promoter exchange studies, strain JABs24 was used to replace the native promoter P_*srfA*_ markerless by either P_*nasD*_ or P_*narG*_ similar to the protocol described by Vahidinasab et al. (2020). Briefly, generated fragments upstream(P_*srfA*_)-P_*narG*_-downstream(P_*srfA*_) and upstream(P_*srfA*_)-P_*nasD*_-downstream(P_*srfA*_) were ligated into SmaI digested plasmid pJOE4786.1 resulting in plasmids pRIK2 for P_*srfA*_::P_*narG*_ and pPB1 for P_*srfA*_::P_*nasD*_. After confirmation by sequencing, fragments of interest were digested of pRIK2 and pPB1 by HindIII and were ligated into plasmid pJOE6743.1 resulting in plasmids for chromosomal promoter exchange, called pRIK4 (P_*srfA*_::P_*narG*_) and pPB2 (P_*srfA*_::P_*nasD*_). These plasmids were isolated and transformed in natural competent *B. subtilis* JABs24 cells. A markerless promoter exchange of P_*srfA*_ was ensured by mannose counterselection as described by Wenzel and Altenbuchner (2015). Final validation of successful integration was performed by sequencing and resulted in strains MG11 for P_*srfA*_::P_*narG*_ and MG13 for P_*srfA*_::P_*nasD*_. Using plasmids pKAM446 (P_*srfA*_-*lacZ*), pKAM452 (P_*narG*_-*lacZ*) and pSHX2 (P_*nasD*_-*lacZ*) for construction of reporter strains and the protocol described in Hoffmann et al. (2020), the integration of promoter-*lacZ* fusion into *amyE* locus was performed for strains JABs24, MG11 and MG13. This resulted in strains KM1016 (*amyE*::[P_*srfA*_-*lacZ, spcR*]), MG12 (P_*srfA*_::P_*narG*_; *amyE*::[P_*narG*_-*lacZ, spcR*]) and MG14 (P_*srfA*_::P_*nasD*_; *amyE*::[P_*nasD*_-*lacZ, spcR*]), respectively.

### Cultivation medium and conditions

Main cultivations were performed in 500 mL baffled shake flasks with relative filling volumes (Rv) of 10% (= 0.1 mL/mL), 50% (= 0.5 mL/mL) and 100% (= 1 mL/mL) at 120 rpm. This approach results in different oxygen availabilities in shake flasks (Heyman et al. 2019; Schiefelbein et al. 2013). In any case, even at 100% Rv, overflowing during shaking did not occur as the shake flask neck posed a sufficient barrier. Cultivations in bioreactors were performed in custom-built bioreactors (ZETA GmbH, Graz/Lieboch, Austria) equipped with foam centrifuge as described in Hoffmann et al. (2020) at 37 °C. Due to the installation and feed-program of the bioreactors, batch and feed media were prepared as g/kg respectively mol/kg.

Preparation of LB medium for the first pre-culture and a modified mineral salt medium for all other shake flask and bioreactor cultivations was performed as described in Hoffmann et al. (2020). The concentrations of the buffer, MgSO_4_∙7H_2_O and trace elements were the same, while the glucose concentration was increased to 40 g/L for shake flask cultivations and 20 g/kg for the bioreactor batch medium. The initial nitrogen source was 0.1 mol/L (or mol/kg) NaNO_3_ and 0.1 mol/L (or mol/kg) (NH_4_)_2_SO_4_, if not indicated otherwise in the results part. For bioreactor cultivations, a feed solution was prepared containing 400 g/kg glucose as well as 20 mL TES/kg and 3.69 g/kg MgSO_4_∙7H_2_O.

Pre-cultures and the main culture were prepared as described in Hoffmann et al. (2020). The second pre-culture was inoculated for 12–16 h. Bioreactor cultivations had an initial batch volume of 20 kg. The agitation rate was fixed at 300 rpm throughout cultivation. A constant aeration rate of 1.2 L/min, equal to 0.06 vvm at T_0_ and the lowest possible aeration rate with the given technical equipment, was set without further pO_2_ regulation. pH was maintained at 7 by addition of 4 M NaOH and 4 M HNO_3_, the latter one serving as self-regulated nitrate-feed due to the basic pH shift caused by anaerobic nitrate respiration. The foam centrifuge run at 2790 rpm when activated by a sensor in the headspace of the bioreactor vessel. The feed was started when glucose became depleting with a set growth rate of 0.03 1/h and an initial feed addition of 0.04 kg/h. The feed was limited to 2 kg.

### Sampling and sample analysis

After measuring the OD_600_ (Biochrom WPA CO8000, Biochrom Ltd., Cambridge, UK) of the samples, cell-free supernatants were stored at – 20 °C. Cell dry weight (CDW) was calculated by dividing the OD_600_ by the factor 3.762, which was established previously (Geissler et al. 2019b). Glucose and surfactin concentrations were determined using an HPTLC method as specified in Geissler et al. (2017, 2019b). Nitrate, nitrite and ammonium concentrations were analyzed with spectrophotometric assays (nitrate: Cat. No. 1.09713.0001, nitrite: Cat. No. 1.14776.0001, ammonium: Cat. No. 1.14752.0001, Merck KGaA, Darmstadt, Germany). Miller units (MU) as indicator for the promoter activity of the *lacZ*-fused promoters were determined by the β-galactosidase assay as described in Hoffmann et al. (2020).

### Data analysis

Process parameters were calculated in the same approach, means Δ*t* yields and overall yields, as explained in Hoffmann et al. (2020). For bioreactor cultivations, all concentrations were converted to absolute values by multiplying the respective value by the weight of the medium. This allowed to compensate for the dilution occurred due to the addition of feed, acid and base solutions. All experiments were performed in duplicates. Depending on the aim of the respective experiment, either strain JABs24, KM1016, MG1 or MG5 was used as reference strain.

## Results

### Influence of different oxygen availabilities on nitrogen metabolism

**Fig. 1:**
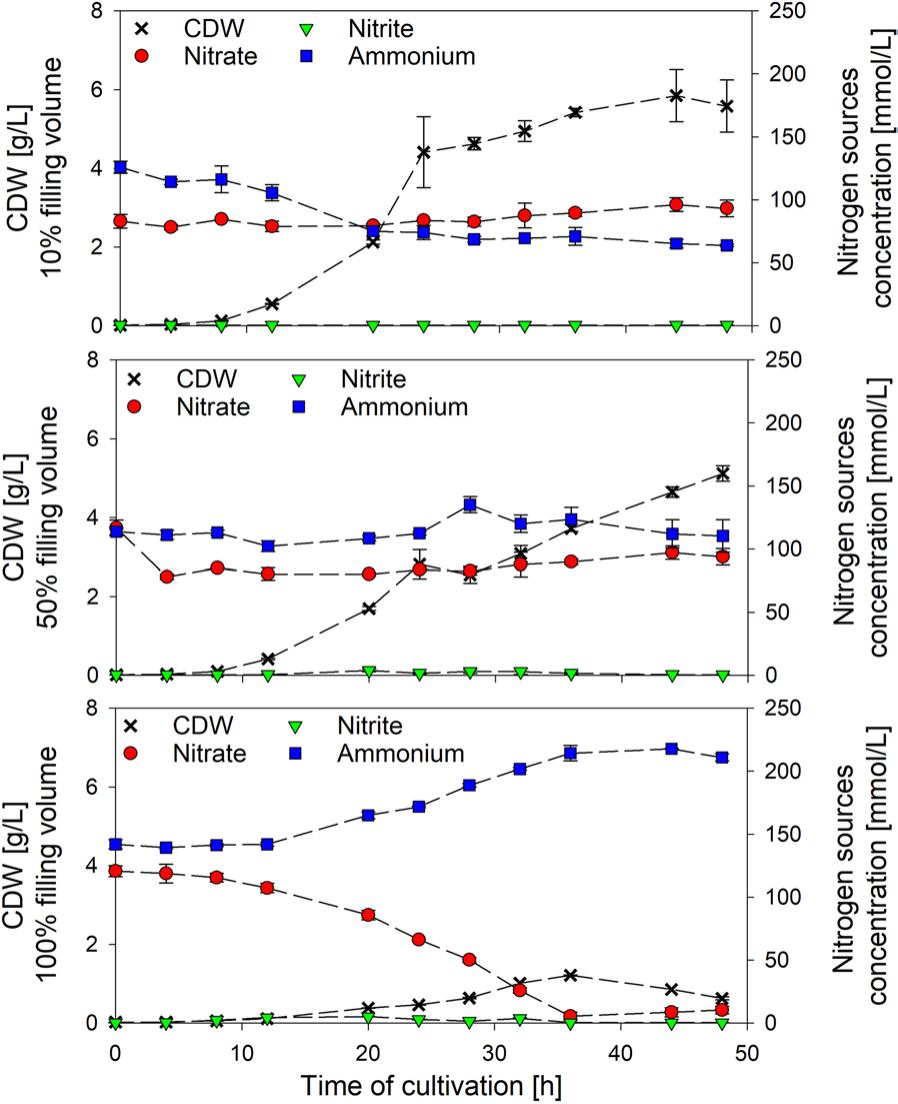
Cell dry weight (CDW) (g/L) and concentrations of nitrate, nitrite and ammonium (all [mmol/L]) of strain *B. subtilis* JABs24 cultivated in a mineral salt medium employing 40 g/L glucose, 0.1 mol/L NH_4_^+^ and 0.1 mol/L NO_3_^−^ in baffled shake flasks with three different relative filling volumes of 10%, 50% and 100% representing different oxygen availabilities

Varying filling volumes in shake flasks are associated with different oxygen availabilities for cells. In case of *B. subtilis*, altered patterns in nitrogen consumption were expected due to assimilatory and dissimilatory pathways. More precisely, low filling volumes result in highest oxygen availability (Heyman et al. 2019; Schiefelbein et al. 2013) and hence aerobic growth is predominant. In this sense, ammonium should be identified as preferred nitrogen source for biomass assimilation, when both ammonium and nitrate are present. High filling volumes result in low oxygen availability (Heyman et al. 2019; Schiefelbein et al. 2013) and nitrate consumption increases due to anaerobic nitrate respiration. To validate these theses, *B. subtilis* JABs24 was cultivated in 10%, 50% and 100% relative filling volume (Rv). Figure 1 displays the corresponding CDW and the concentrations of nitrate, nitrite and ammonium during the time course of cultivation. CDW_max_ was reached after 44 h, 48 h and 36 h with 5.85 ± 0.66 g/L, 5.12 ± 0.20 g/L and 1.21 ± 0.01 g/L at increasing cultivation volumes. Additional glucose measurements excluded carbon limitation (data not shown). Nitrate concentrations were relatively constant at 10% and 50% Rv, with a slight decrease detectable in the last-mentioned. On the contrary, nitrate limitation occurred in 100% Rv after 36 h. Hence, growth was limited by nitrate depletion. As expected, the ammonium concentration decreased during cultivation with 10% Rv. At 50% Rv, ammonium initially decreased slightly before a relatively constant concentration could be measured. A continuous increase in ammonium was detected in the cultivation using 100% Rv of the flask capacity. Nitrite, the intermediate of nitrate respiration, was not detected in cultivations using 10% Rv. Nitrite peaked to values of 4.11 ± 1.17 mmol/L and 4.95 ± 0.03 mmol/L after 20 h of cultivation in both 50% and 100% Rv, respectively. Those nitrite concentrations particularly affect a prolonged lag phase of cultivation of anaerobically growing *B. subtilis* cultures, while the values of maximum specific growth rates µ_max_ remain unchanged (Hoffmann et al. 2020). However, concentrations further declined until CDW_max_. Hence, the time course of nitrogen sources indicated that higher filling volumes resulted in a converged nitrogen consumption pattern. More precisely, nitrate respiration was preferred with increasing Rv and hence lower oxygen availabilities can be assumed.

### Suitability of the promoters P_*narG*_ and P_*nasD*_ for gene expression under anaerobic conditions

**Fig. 2:**
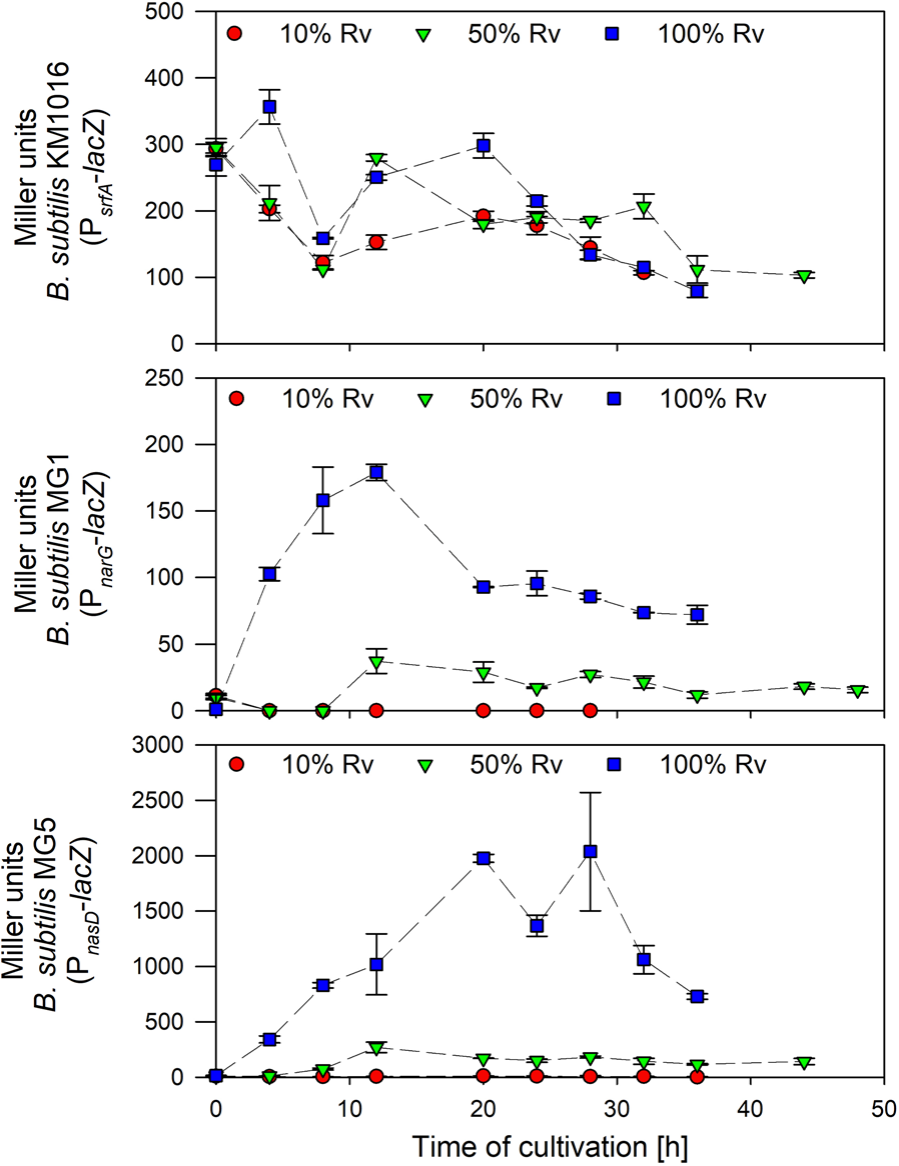
Time course of Miller units determined by the Miller Assay representing expression of promoters P_*srfA*_, P_*narG*_ and P_*nasD*_. Data recorded until CDW_max_ of cultivation of strains *B. subtilis* KM1016 (P_*srfA*_-*lacZ*), MG1 (P_*narG*_-*lacZ*) and MG5 (P_*nasD*_-*lacZ*) in a mineral salt medium employing 40 g/L glucose, 0.1 mol/L NH_4_^+^ and 0.1 mol/L NO_3_^−^ in baffled shake flasks with three different relative filling volumes (Rv) of 10%, 50% and 100% representing different oxygen availabilities

The nitrogen patterns of the preliminary cultivations have indicated that different oxygen availabilities are present in cultivations with 10%, 50% and 100% Rv. Further cultivations aimed at investigating the suitability and expression of P_*narG*_ and P_*nasD*_ as anaerobically inducible promoters. Therefore, strains KM1016, MG1 and MG5 carrying P_*srfA*_-*lacZ*, P_*narG*_-*lacZ* and P_*nasD*_-*lacZ*-fusions, respectively, were cultivated in shake flasks with 10%, 50% and 100% Rv. Comparable growth curves were observed as described in Fig. 1. Figure 2 displays the promoter activities as determined by Miller units (MU) during cultivations.

The P_*srfA*_ expression was comparatively congruent amongst the different Rv values tested. Altogether, a marginal tendency to higher mean MU with increasing Rv was noticed. In general, P_*srfA*_ expression dropped from ~ 300 MU to ~ 125 MU after 8 h of cultivation. Another slight increase in expression was followed by a subsequent decline to 70–100 MU at CDW_max_. For both P_*narG*_ and P_*nasD*_ expressions, MU increased with increasing Rv. At 10% Rv, LacZ activity was not detectable for transcriptional fusion with P_*narG*_ and expression did not surpass 10 MU for P_*nasD*_. At 50% Rv, both expression levels of P_*narG*_ and P_*nasD*_ increased to a maximum after 12 h of cultivation with ~ 40 MU and ~ 270 MU, respectively, with an ensuing decrease to ~ 15 MU and ~ 140 MU until CDW_max_. Highest expression levels were monitored at 100% Rv. For P_*narG*_, expression increased to ~ 180 MU after 12 h of cultivation before a decline to ~ 80 MU was detectable. In contrast to that, maximum expression of P_*nasD*_ was ~ 10-fold higher and reached ~ 2000 MU after 20 h of cultivation. Noticeably, comparable to P_*narG*_ expression, a reduction of MU values to ~ 800 MU could be observed at CDW_max_. In this context, as already described by Hoffmann et al. (2020), a strong P_*nasD*_ activity is detectable, although the overflow metabolite acetate increases with higher glucose amounts in anaerobic cultivations. In summary, while P_*srfA*_ expression was relatively constant amongst the different Rv tested, both P_*narG*_ and P_*nasD*_ expressions were induced at higher Rv and hence lower oxygen availabilities. More specifically, P_*nasD*_ revealed the overall highest expression values.

To apply P_*narG*_ and P_*nasD*_ promoter systems for bioprocesses, more information about *B. subtilis* physiology is important. In brief, *B. subtilis* harbors two nitrate reductases with the genes *nasBC* and *narGHI* encoding for the assimilatory and dissimilatory nitrate reductase, respectively. The transcription of the latter one, *narGHI*, is thereby repressed in the presence of oxygen (Hoffmann et al. 1995; Nakano et al. 1996). On the contrary, *nasDE* encodes for both assimilatory and dissimilatory nitrite reductase and transcription is hence also feasible aerobically (Nakano et al. 1998). To demonstrate that the P_*nasD*_ expression is dependent on the presence of ammonium or nitrate, respectively, *B. subtilis* MG5 was cultivated in media containing either NH_4_^+^ or NO_3_^−^ in 10% Rv. As expected, mean MU for P_*nasD*_ expression was below 10 MU in the medium containing only NH_4_^+^, whereas much higher MU values (continuously ~ 200 MU) were detected in NO_3_^−^ supplemented medium (Additional file 1: Fig. S1). In addition, in media supplemented with either 0.005 or 0.01 mol/L NH_4_^+^, a time-delayed expression of P_*nasD*_ was monitored (Additional file 1: Fig. S1) which was triggered by depletion of NH_4_^+^ (data not shown). Hence, it can be concluded that P_*nasD*_ is a strong, anaerobically inducible promoter, but the effect of anaerobic induction is dependent on the availability of NH_4_^+^ during aerobic growth.

### Investigation of strains *B. subtilis* MG12 and MG14 carrying promoter exchanges P_*srfA*_::P_*narG*_ and P_*srfA*_::P_*nasD*_

#### Growth behavior and surfactin synthesis

**Fig. 3:**
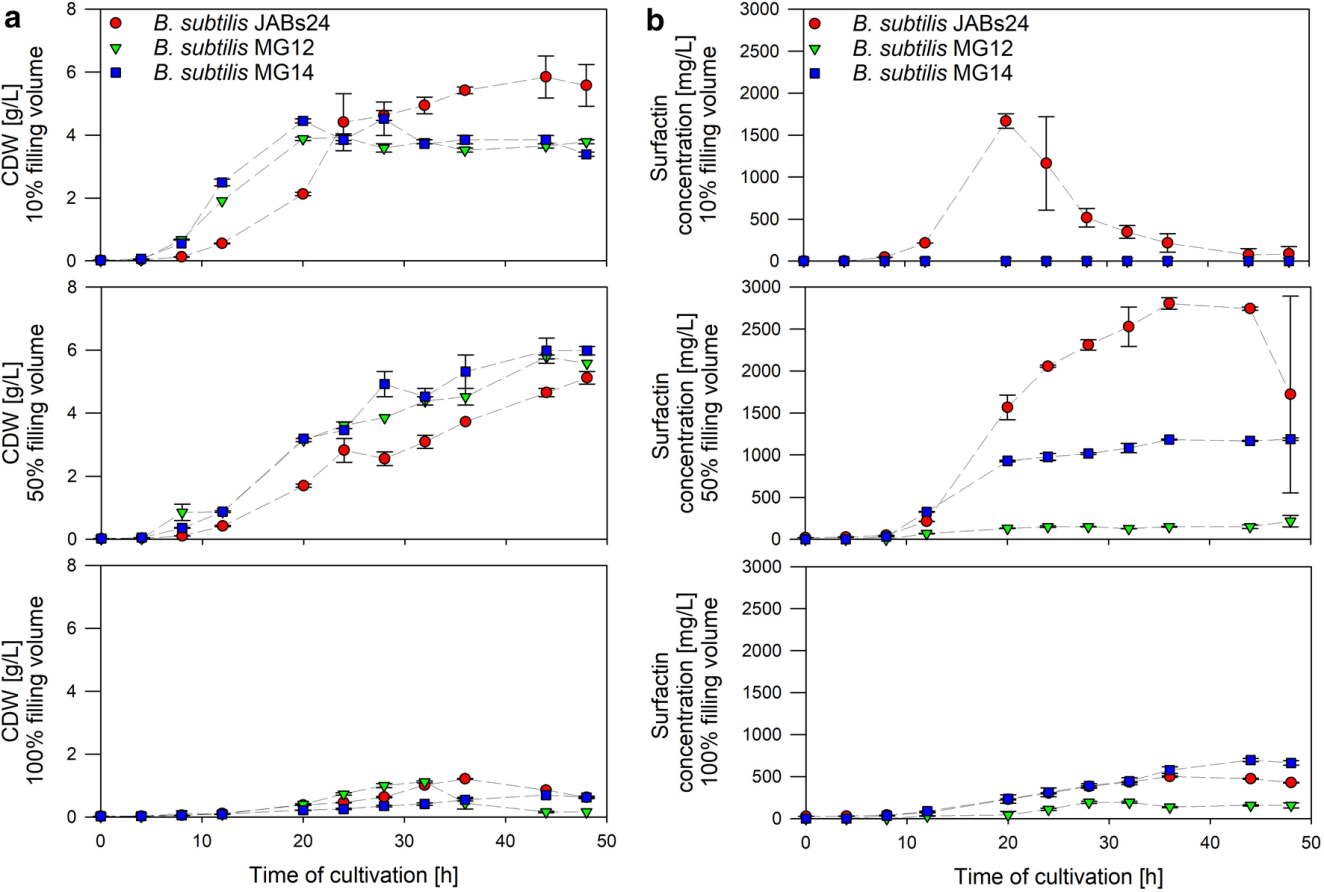
Time course of cell dry weight (CDW) [g/L] (**a**) and surfactin concentration [mg/L] (**b**) of strains *B. subtilis* JABs24 (reference), MG12 (P_*narG*_-*lacZ; amyE*::[P_*narG*_-*lacZ, spcR*]) and MG14 (P_*srfA*_::P_*nasD*_; *amyE*::[P_*nasD*_-*lacZ, spcR*]) cultivated in a mineral salt medium with 40 g/L glucose, 0.1 mol/L NH_4_^+^ and 0.1 mol/L NO_3_^−^ employing three different relative filling volumes of 10%, 50 and 100% representing different oxygen availabilities

The promoter exchange strains MG12 (P_*srfAA*_::P_*narG*_; *amyE*::[P_*narG*_-*lacZ, spcR*]) and MG14 (P_*srfA*_::P_*nasD*_; *amyE*::[P_*nasD*_-*lacZ, spcR*]) were cultivated in shake flasks with 10%, 50% and 100% Rv. An overview of CDW and surfactin concentrations of these strains, as well as the reference strain JABs24, are displayed in Fig. 3a and b. In 10% Rv, strains MG12 and MG14 reached CDW_max_ after 24 h and 20 h with 3.93 ± 0.11 g/L and 4.45 ± 0.07 g/L, respectively. CDW remained constant until the end of cultivation. CDW_max_ for strain JABs24 was reached after 44 h with 5.85 ± 0.66 g/L. In 50% Rv, all strains reached CDW_max_ after 44 to 48 h with 5.12 ± 0.20 g/L, 5.78 ± 0.07 g/L and 5.98 ± 0.30 g/L for strains JABs24, MG12 and MG14, respectively. In contrast, CDW_max_ was much lower in cultivations with 100% Rv and did not surpass 1.21 ± 0.01 g/L, 1.12 ± 0.03 g/L and 0.69 ± 0.00 g/L for strains JABs24, MG12 and MG14 after 36 h, 32 h and 44 h, respectively, before the concentration of biomass dropped. In addition, cell agglutination was visible with progressive cultivation causing difficulties in OD_600_ measurements. However, strong induction of P_*nasD*_ activity was detected, suggesting that cell agglutination does not counteract *nasD* expression.

As expected, strains MG12 and MG14 did not synthesize surfactin at 10% Rv (Fig. 3b). For the reference strain JABs24, the maximum surfactin concentration was measured after 20 h of cultivation with 1668 ± 87 mg/L. During subsequent bacterial growth, the concentration of surfactin declined to 146 ± 118 mg/L at CDW_max_. The surfactin concentration of the reference strain was even higher with 2806 ± 68 mg/L in 50% Rv. Under this condition, strain MG12 produced up to 215 ± 69 mg/L and strain MG14 up to 1189 ± 15 mg/L. In 100% Rv, lowest surfactin titer was detected for strain MG12 with 193 mg/L, surfactin_max_ of strain MG14 surpassed the titer of strain JABs24 by ~ 200 mg/L with 696 ± 19 mg/L. With respect to foam formation, this issue could be observed for strain JABs24 from the beginning in 10% and 50% Rv, while foam formation was time-delayed for strains MG12 and MG14. At 100% Rv, foam was not present in any cultivation due to the high fluid level and low turbulences.

The corresponding Miller units of strains MG12 and MG14 are displayed in Additional file 1: Fig. S2. In sum, MU values of strain MG12 were similar to strain MG1 (Fig. 2). For strain MG14, MU values in 10% and 50% Rv were also comparable to the cultivation of strain MG5 (Fig. 2). At 100% Rv, highest MU values for MG14 did not surpass ~ 1100 MU and remained relatively constant until CDW_max_.

In all cultivations, glucose was not depleted. With respect to the nitrogen sources, displayed in Additional file 1: Fig. S3, the patterns were similar as illustrated in Fig. 1 with one exception. In 50% Rv, nitrate consumption and hence increase in ammonium were more conspicuous in strains MG12 and MG14 than for the reference strain. After ~ 32 h of cultivation, nitrate was almost depleted and until this time point ammonium increased.

#### Strain *B. subtilis* MG14 reaches remarkable *Y*_P/X_ values

All yields and growth rates of the cultivation in 10%, 50% and 100% Rv are displayed in Table 2. *B. subtilis* MG12 reached highest overall and maximum growth rates in all cultivations. In 10% and 50% Rv, JABs24 had lowest overall and maximum growth rates. Using 100% Rv, however, lowest growth rates were monitored for strain MG14. A trend amongst the strains for the *Y*_X/S_ was not identified. Highest Rv and hence lowest oxygen availability resulted in lowest *Y*_X/S_. As no surfactin was produced in 10% Rv for strain MG12 and MG14, no data could be calculated for *Y*_P/X_, *Y*_P/S_ and *q*. In accordance with surfactin concentrations, strain JABs24 was superior with respect to *Y*_P/X_, *Y*_P/S_ and *q* in cultivations using 50% Rv. Noticeable, strain MG14 reached an overall *Y*_P/X_ of 1.007 ± 0.028 g/g in 100% Rv, representing an excellent result. In addition, the overall and maximum productivity *q* was the highest for strain MG14 in 100% Rv with 0.023 ± 0.001 g/(g h) and 0.209 ± 0.012 g/(g h), even surpassing the productivities of strain JABs24 in 10% and 50% Rv.

**Table 2:** Comparison of calculated overall and ∆*t*_max_ yields and specific growth rates *µ* of shake flask cultivations with different relative filling volumes employing strains *B. subtilis* JABs24 (reference), MG12 (P_*srfA*_::P_*narG*_ *amyE*::[P_*narG*_-*lacZ*, *spcR*]) and MG14 (P_*srfA*_::P_*nasD*_ *amyE*::[P_*nasD*_-*lacZ*, *spcR*])

Relative filling volume	*B. subtilis* strain	Overall yield to CDW_max_				
		*Y*_P/X_ [g/g]	*Y*_X/S_ [g/g]	*Y*_P/S_ [g/g]	*q* [g/(g h)]	*µ* [1/h]
10%	JABs24	0.021 ± 0.001	0.216 ± 0.026	0.005 ± 0.001	0.001 ± 0.000	0.144 ± 0.013
	MG12	0.000 ± 0.000	0.175 ± 0.030	0.000 ± 0.000	0.000 ± 0.000	0.223 ± 0.001
	MG14	0.000 ± 0.000	0.239 ± 0.055	0.000 ± 0.000	0.000 ± 0.000	0.185 ± 0.002
50%	JABs24	0.346 ± 0.242	0.129 ± 0.000	0.044 ± 0.031	0.007 ± 0.005	0.116 ± 0.002
	MG12	0.026 ± 0.004	0.183 ± 0.012	0.005 ± 0.001	0.001 ± 0.000	0.130 ± 0.000
	MG14	0.191 ± 0.009	0.181 ± 0.019	0.035 ± 0.002	0.004 ± 0.000	0.122 ± 0.005
100%	JABs24	0.413 ± 0.002	0.090 ± 0.006	0.036 ± 0.002	0.011 ± 0.000	0.111 ± 0.001
	MG12	0.173 ± 0.015	0.120 ± 0.064	0.020 ± 0.010	0.005 ± 0.000	0.126 ± 0.003
	MG14	1.007 ± 0.028	0.051 ± 0.006	0.053 ± 0.005	0.023 ± 0.001	0.079 ± 0.000

Relative filling volume	*B. subtilis* strain	∆*t*_max_				
		*Y*_P/X_ [g/g]	*Y*_X/S_ [g/g]	*Y*_P/S_ [g/g]	*q* [g/(g h)]	*µ* [1/h]
10%	JABs24	1.081 ± 0.039	0.535 ± 0.181	0.194 ± 0.001	0.141 ± 0.008	0.369 ± 0.022
	MG12	0.000 ± 0.000	0.289 ± 0.032	0.000 ± 0.000	0.000 ± 0.000	0.775 ± 0.006
	MG14	0.000 ± 0.000	0.339 ± 0.093	0.000 ± 0.000	0.000 ± 0.000	0.527 ± 0.005
50%	JABs24	1.273 ± 0.095	0.629 ± 0.156	0.217 ± 0.037	0.169 ± 0.002	0.343 ± 0.001
	MG12	0.080 ± 0.007	0.331 ± 0.059	0.304 ± 0.279	0.020 ± 0.002	0.833 ± 0.078
	MG14	0.476 ± 0.005	0.451 ± 0.130	0.629 ± 0.554	0.119 ± 0.001	0.471 ± 0.003
100%	JABs24	0.642 ± 0.015	0.208 ± 0.015	0.099 ± 0.017	0.090 ± 0.001	0.205 ± 0.007
	MG12	0.303 ± 0.030	0.291 ± 0.036	0.030 ± 0.012	0.076 ± 0.008	0.388 ± 0.015
	MG14	1.061 ± 0.179	0.161 ± 0.089	0.243 ± 0.135	0.209 ± 0.012	0.151 ± 0.002

### Bioreactor cultivations

As a rule of thumb, filling volume in shake flasks should not exceed 10% (Rv = 0.1 mL/mL) of flask nominal volume to ensure sufficient oxygen supply. However, the previous shake flask cultivations have demonstrated that reference strain JABs24 reached highest surfactin titer at a 50% Rv, and even at 100% Rv both *Y*_P/X_ and *q* were promising. In the latter condition, *B. subtilis* MG14 with P_*srfA*_::P_*nasD*_ even surpassed the surfactin titers, *Y*_P/X_ and *q* of the reference strain. Consequently, the next step aimed at transferring these results to bioreactor scale.

### Reference process and preliminary pO_2_ strategies

CDW, surfactin and pO_2_ levels of different processes employing *B. subtilis* JABs24 or MG1 are given in Fig. S4. A conventional batch process with foam centrifuge employing 20 g/kg glucose was performed as control with an initial agitation and aeration rate of 300 rpm and 2.0 L/min and a pO_2_ set-point of 20% (Additional file 1: Fig. S4a). CDW_max_ was reached after 24 h with 77.51 g prior to glucose depletion. Simultaneously, surfactin_max_ was reached with 57.43 g. Nitrate was almost constant throughout cultivation, while ammonium was reduced by ~ 50%. In addition, slight persistent foaming occurred which was accompanied by an increase in the pressure due to blocked exhaust air filters. In a subsequent fed-batch approach, the aeration rate was stopped after 21 h of cultivation and switched to N_2_ based ventilation (Additional file 1: Fig. S4b). At this time point, the pO_2_ was already at 20% for ~ 5 h. When the process air was stopped, the pO_2_ dropped to 0% within seconds. However, also the CDW dropped from 50.27 g to 3.67 g within 4.5 h. Surfactin reached 29.48 g prior to the change in pO_2_ and was reduced slightly when CDW dropped. However, cells restored the growth and after 41.5 h of cultivation, a biomass of 24.1 g was obtained with a slight increase in surfactin to 32 g. In a next approach, the pO_2_ was decreased stepwise to ideally avoid the observed drop of CDW by cell adaptation (Fig. S4c). Each pO_2_ level of 20%, 10%, 5% and 0% was kept for 3 h before aeration was switched to N_2_. In this approach, the CDW as well as surfactin still dropped, but to a much lower extent, from 80.74 g to 61.75 g, and 39.84 g to 34.80 g, respectively. Both CDW and surfactin increased afterwards and reached maximum values of 73.26 g and 41.91 g.

### Comparison of strains *B. subtilis* KM1016 and MG14 with 1.2 L/min aeration rate employing a self-regulated HNO_3_ feed

**Fig. 4:**
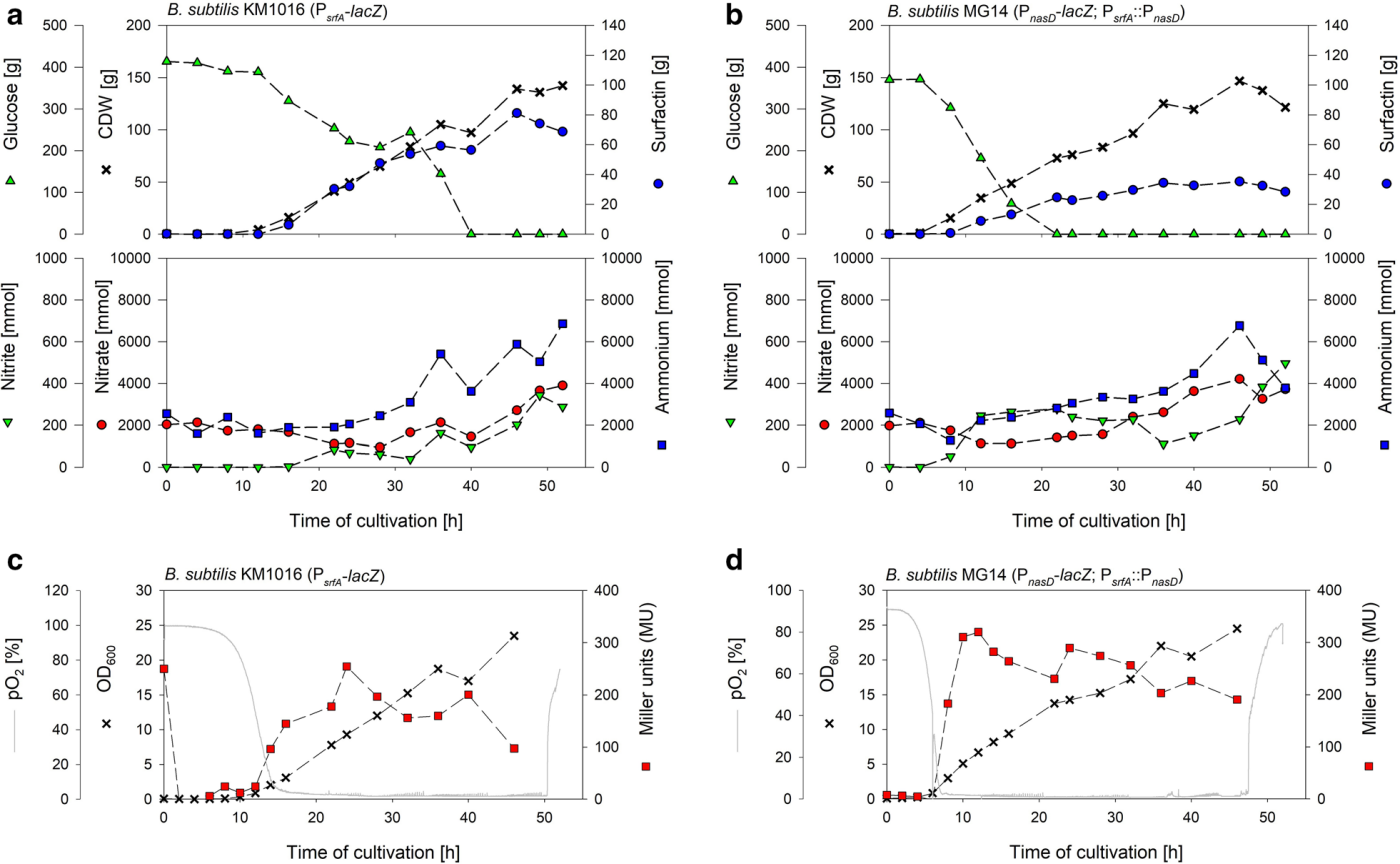
Exemplary cultivation results of fed-batch bioreactor cultivations employing strains *B. subtilis* KM1016 (P_*srfA*_-*lacZ*) (**a**, **c**) and *B. subtilis* MG14 (P_*srfA*_::P_*nasD*_; *amyE*::[P_*nasD*_-*lacZ, spcR*]) (**b**, **d**). Displayed are absolute values of cell dry weight (CDW), glucose, surfactin (all [g]), nitrate, nitrite and ammonium (all [mmol]) (**a**, **b**), as well as the time course of OD_600_, Miller units as determined by the Miller Assay to determine the expression of P_*srfA*_ and P_*nasD*_, and pO_2_ [%] (**c**, **d**)

Due to the observed drops in CDW and the concomitant stagnating surfactin production, another approach was tested employing a constant aeration and agitation rate throughout the cultivation. This should result in a decline of available oxygen per cell and hence an adaptation of cells to microaerobic conditions. First, a fed-batch cultivation was performed with a constant aeration rate of 2 L/min employing the reporter strain MG1 to monitor P_*narG*_ expression, which is exclusively activated anaerobically. Cells grew without drop and reached a CDW_max_ of 196 g after 40 h. Surfactin values increased constantly during growth to a maximum of 104 g (Fig. S4d). Miller units revealed that only a small portion of cells adapted to anaerobic conditions. During Miller Assay, the yellow color as indicator to stop the reaction was visible in less than 3 min, but calculation revealed 5 MU, as the high OD_600_ of 30 is included in the calculation. Hence, it can be assumed that a high expression level of the promoter is present, but most cells did not grow by nitrate respiration. A cultivation with 1.2 L/min and strain MG1, which actually represents the lowest aeration rate that could be set with the given technical equipment, resulted in Miller units up to 35 for P_*narG*_ and was hence used as process to compare *B. subtilis* KM1016 and MG14 with P_*srfA*_::P_*nasD*_. Figure 4 exemplarily illustrates the CDW, glucose, surfactin, nitrate, nitrite and ammonium values of a cultivation with *B. subtilis* KM1016 (Fig. 4a) and *B. subtilis* MG14 (Fig. 4b). In addition, OD_600_ in correlation to Miller units and pO_2_ are given in Fig. 4c and d for strains KM1016 and MG14, respectively. Comparable CDW_max_ values were reached for strain KM1016 and MG14 after 46 h with 140 g and 147 g, respectively, whereas the growth was terminated by glucose depletion. In contrast, different surfactin_max_ were reached at CDW_max_ with 81 g for KM1016 and 35 g for MG14. Strain KM1016 consumed a total of 5.6 mol nitrate until CDW_max_. Nitrate addition by HNO_3_ was higher than consumption, resulting in a nitrate accumulation to 2.7 mol in the medium. Strain MG14 showed a similar behavior but consumed less nitrate. At CDW_max_, total nitrate consumption was 4.5 mol and another 4.2 mol were detectable in the medium.

The detection of ammonium showed an initial slight decrease before the amount increased steadily for both strains due to nitrate reduction and reached 5.8 mol and 6.7 mol for strains KM1016 and MG14, respectively. Nitrite concentrations increased until 22 h in both cultivations. After a subsequent decrease until ~ 30 h, nitrite again increased steadily and reached values of ~ 0.21 mol at CDW_max_. The pO_2_ declined faster in the cultivation of strain MG14 which correlated to higher growth rates (Fig. 4d). For strain KM1016, a pO_2_ of ~ 14% was reached after ~ 15 h, and mean pO_2_ of ~ 1.8% was measured after 25 h of cultivation (Fig. 4c). On the contrary, the pO_2_ of strain MG14 fell to ~ 2% after 7 h of cultivation, and a mean pO_2_ of ~ 1.2% was reached after ~ 15 h of cultivation. For both strains, the pO_2_ increased drastically when cell growth was terminated. In terms of foaming, the foam centrifuge was activated right from the beginning for strain KM1016 and after ~ 6 h for strain MG14. With respect to the Miller units, P_*srfA*_ expression was detectable as soon as cells grew after a short lag-phase. A maximum of 250 MU was determined after 24 h of cultivation and values slightly decreased afterwards (Fig. 4c). For P_*nasD*_, induction levels were below 5 MU until 6 h of cultivation. In accordance with the pO_2_, induction increased to 180 MU after 8 h when the pO_2_ was < 2%. Highest expression was detected after 12 h with 320 MU before expression declined slightly (Fig. 4d).

Table 3 summarizes the main parameters and overall yields of the cultivations with strains KM1016 and MG14 employing an aeration rate of 1.2 L/min. For comparison, data of the reference process with *B. subtilis* JABs24 and the processes employing a constant aeration rate of 2 L/min with strain MG1 are included. Corresponding calculated Δ*t*_max_ yields and growth rates are listed in Additional file 1: Table S3. For fed-batch processes and for better comparison to the reference batch cultivation, results are displayed as “after batch glucose consumption” calculations and “end of process” calculations. The fed-batch process employing the reference strain and 1.2 L/min reached only 82.14 g of surfactin, which resulted in a productivity *q* of 0.012 g/(g h). This value is similar to the 2 L/min fed-batch process with 0.013 g/(g h), where 104.57 g of surfactin were produced, but with biomass of 196.97 g. Comparing batch data, the reference batch process had a productivity *q* of 0.031 g/(g h). Here, the 2 L/min process was inferior with 0.022 g/(g h) and the 1.2 L/min process performed similar with 0.030 g/(g h). Comparing strains KM1016 and MG14, both processes reached comparable CDW of 147.52 g and 150.18 g, but the surfactin produced with strain MG14 with 31.56 g was ~ 2.5-fold lower. Hence, both productivities and yields were inferior for strain MG14 and solely the growth rates during the batch phase were superior.

**Table 3:** Summary of main process data, calculated overall yields and specific growth rates of *B. subtilis* bioreactor cultivations

Strategy	Reference pO_2_ set-point 20%	Fed-batch, constant aeration of 2 L/min		Fed-batch, constant aeration of 1.2 L/min		Fed-batch, constant aeration of 1.2 L/min	
Evaluation until	End of batch	After batch glucose consumption	End of process	After batch glucose consumption	End of process	After batch glucose consumption	End of process
*B. subtilis* strain	JABs24	MG1		KM1016		MG14	
Time of cultivation (h)	24	22	40	26	46	19	46
CDW_max_ (g)	77.51	104.55	196.97	61.54 ± 9.21	147.52 ± 8.22	55.78 ± 13.24	150.18 ± 3.33
Surfactin_max_ (g)	57.43	51.65	104.57	48.47 ± 11.81	82.14 ± 0.91	15.49 ± 6.40	31.56 ± 3.74
*Y*_P/X_ (g/g)	0.741	0.494	0.531	0.776 ± 0.088	0.557 ± 0.024	0.266 ± 0.039	0.211 ± 0.030
*Y*_X/S_ (g/g)	0.145	0.249	0.195	0.161 ± 0.011	0.131 ± 0.005	0.134 ± 0.016	0.123 ± 0.003
*Y*_P/S_ (g/g)	0.108	0.124	0.103	0.127 ± 0.022	0.073 ± 0.001	0.036 ± 0.010	0.026 ± 0.004
*q* (g/(g∙h))	0.031	0.022	0.013	0.030 ± 0.003	0.012 ± 0.001	0.014 ± 0.002	0.004 ± 0.001
*µ* (1/h)	0.222	0.272	0.165	0.204 ± 0.009	0.134 ± 0.005	0.271 ± 0.010	0.128 ± 0.003

## Discussion

The current study aimed at introducing a promoter exchange strain that allows for self-inducible surfactin synthesis when oxygen-limiting, or even depleting, conditions occur and anaerobic nitrate respiration initiates. To mimic different oxygen availabilities in shake flask cultivations, either the filling volume or the agitation rate can be varied. This method was already used in other studies investigating surfactin synthesis in *B. subtilis* (Fahim et al. 2012; Jokari et al. 2013; Rangarajan et al. 2015; Rocha et al. 2020). In the current study, different filling volumes were employed. Studies of Schiefelbein et al. (2013) and Heyman et al. (2019), investigating either the kLa value or oxygen transfer rate OTR, have demonstrated that higher filling volumes result in lower oxygen availability. Results from this study supported the hypothesis when nitrogen sources were analyzed at different Rv resulting in either aerobic growth on ammonium as preferred nitrogen source, or anaerobic growth by nitrate respiration.

Experiments have demonstrated that *B. subtilis* MG14 with P_*srfA*_::P_*nasD*_ poses an interesting candidate for further bioreactor process developments targeting at self-inducible surfactin synthesis uncoupled from the native quorum-sensing system. In addition, this self-inducible system is advantageous compared to IPTG-induced promoters due to reduced production costs. Furthermore, foaming was also lowered, and time delayed in both shake flask and bioreactor cultivations. Interestingly, both strains MG12 (P_*srfA*_::P_*narG*_) and MG14 showed improved growth rates when no surfactin was produced in 10% Rv. At both 50% and 100% Rv, increased growth rates were obtained with decreasing surfactin titers. Hence, expression and presence of surfactin seems to result in reduced growth rates, which is in accordance with previous studies (Tsuge et al. 2001; Vahidinasab et al. 2020). Shake flask cultivations employing different Rv have been shown to be a suitable approach to investigate the activation of the promoters P_*narG*_ and P_*nasD*_. In the presented set-up, the reference strains MG1 and MG5 reached Miller units of up to 2000 for P_*nasD*_ and 180 for P_*narG*_, respectively. Hence, P_*nasD*_ expressions were even slightly higher than reported by Hoffmann et al. (2020) when cultivating in anaerobic serum flasks. On the contrary, P_*narG*_ expression levels were reported to reach up to 440 MU (Hoffmann et al. 2020). Consequently, as P_*narG*_ is an exclusively anaerobically activated promoter, the combination of 100% Rv at 120 rpm probably did not result in full anaerobic induction. One possibility to further decrease the oxygen input would be to vary the agitation rate as performed by Fahim et al. (2012). However, cell agglutination already occurred at 120 rpm and is expected to appear even earlier at lower agitation rates. Hence, as nitrate was often observed as growth limiting factor, increasing the nitrate concentration to elongate the cultivation would not be possible under these circumstances. One option would be to perform further strain engineering and delete genes responsible for biofilm formation and thus for cell agglutination (Pedrido et al. 2013). Assuming microaerobic conditions at 100% Rv and 120 rpm, strain MG14 reached up to ~ 3-fold higher P_*nasD*_ expression than P_*srfA*_ in the reference strain. On the contrary, the surfactin concentration was only 1.4-fold increased. Feedback mechanisms or nutrient limitations might result in this observed discrepancy, and further studies must investigate which optimizations have to be performed on a molecular level or in operational parameter to reach a 3-fold increase in surfactin titer to exploit the complete potential of P_*nasD*_.

As reported by Hoffmann et al. (2020), high ammonium and glucose concentrations reduced expression of P_*narG*_, but such a reductive effect was not observed for P_*nasD*_. Still, fine-tuning of the medium is important to find a good balance between sufficient ammonium for aerobically growing cells, and cells performing nitrate respiration, hence providing ammonium. Particularly, the effect of different nitrate concentrations on the expression of both P_*narG*_ and P_*nasD*_ has not been examined so far. This is also of utmost importance when employing a self-regulated HNO_3_-feed as pH regulator. Indeed, the amount of HNO_3_ added was higher than nitrate consumption due to the production of various metabolites that alkalize the medium on top to the pH shift caused by nitrate respiration, resulting in a continuous increase in nitrate. In addition, the amount of HNO_3_ needed is three-times higher than for e.g. H_3_PO_4_ (monovalent vs. trivalent acid). This results in further limitations as the bioreactor capacity is exhausted earlier. With the current set-up it cannot be examined if both oxygen-depleted and nitrate-depleted conditions reach even higher *Y*_P/X_ values as reported by Davis et al. (1999). For this purpose, mathematical modeling of the bioprocess combining a metabolic model of the nitrogen metabolism, oxygen availability and consumption, along with mechanisms of genetic regulation can be used to provide further insights into this complex interplay. Using this toolset, investigation and evaluation of a dual-limitation fed-batch process becomes feasible, which is a very demanding task for process control, as both limitations need to be tightly regulated (Noll and Henkel 2020).

With respect to bioreactor cultivations, the original intention was to perform so called switch-processes, with the first part aiming at biomass accumulation, and the second part resulting in surfactin production. However, a drastic decrease in biomass was observed when cells entered oxygen-limited or even depleted conditions too fast. It was recently reported that ~ 90% of cells died upon oxygen depletion, while the remaining cells maintain their viability (Arjes et al. 2020). Similar observations were made in the current employed processes with drop in CDW. Although cells restored their growth, the surfactin productivity of these cells was reduced. Consequently, when targeting at switch processes with different pO_2_-profiles, it is of utmost importance to relate oxygen availability to adaptation of cells to anaerobic conditions. For example, Hoffmann et al. (1995) reported on a lag-phase of 24 to 36 h when aerobically growing cells were transferred to anaerobic conditions. This would result in time-consuming processes and the increased growth rates observed in aerobic non-surfactin producing cells would probably not compensate for these adaptation times. In a further approach, a cultivation with a constant agitation rate of 300 rpm and aeration rate of 1.2 L/min was tested. This process set-up was identified as easily operable and issues due to foam formation were drastically reduced compared to conventional batch and fed-batch processes with varying agitation and aeration rates due to pO_2_ regulation. In addition, foaming occurred time-delayed in strain MG14 compared to the reference strain KM1016 which can be attributed in parts to the absence of surfactin during the first hours of cultivation. However, foam formation was yet present prior to the induction of surfactin production. In this sense, identification of other metabolites, such as proteins, in foam and deletion of corresponding genes might be a target in future works to further reduce the foam formation. Results of both surfactin titer and promoter activity of P_*nasD*_ also clearly indicated that the employed set-up more likely correlated to shake flask cultivations with 50% Rv. Also based on the Miller units obtained, it can be assumed that most cells still grew aerobically. This leads to the open question how many cells have already adapted to anaerobic conditions and it is crucial to further characterize the cell differentiation patterns in aerobically-anaerobically growing cells. Hence, further bioreactor cultivations must be performed with even lower aeration rates, either by applying air with a lower oxygen concentration (currently 16%), pure oxygen or, of course, by adjusting the technical equipment to allow for aeration rates below 1.2 L/min. Ideally, applying pure oxygen at much lower aeration rates (< 0.2 L/min) would result in two advantages. First, even less foam formation is expected to occur, and second, the autoinduction of surfactin synthesis is expected to be much higher as the available amount of oxygen per cell decreases faster. Another issue faced was the drastic accumulation of nitrite in bioreactor cultivations, which was not observed in shake flasks. At these concentrations, nitrite is reported to reduce and even inhibit cell growth (Hoffmann et al. 2020). Prior to further bioreactor process optimization, studies must address this limitation and investigate the defect of nitrite reduction to ammonium. Nevertheless, even under the current bioreactor process conditions, strain MG14 with a *Y*_P/X_ of 0.211 g/g and productivity *q* of 0.004 g/(g h) was already superior to other non-conventional bioreactor processes reported by Chtioui et al. (2012) (Rotating disc bioreactor, *Y*_P/X_ = 0.068 g/g, *q* = 0.001 g/(g h)) Coutte et al. (2010b) (Membrane bioreactor, *Y*_P/X_ = 0.078 g/g, *q* = 0.002 g/(g h)). A comparative table was prepared previously in Hoffmann et al. (2020). A foam-free anaerobic cultivation was reported to reach a promising *Y*_P/X_ with 0.278 g/g (Willenbacher et al. 2015), but as summarized by Hoffmann et al. (2020), complete anaerobic processes are time-consuming due to low growth rates and the overall surfactin titers are by far not competitive to conventional aerobic batch processes. With further appropriate regulation of the nitrogen sources and aeration, processes employing strain MG14 are expected to become superior to the reference strain JABs24 as demonstrated in shake flask cultivations with a *Y*_P/X_ of 1.007 g/g and productivity *q* of 0.023 g/(g h). Reaching these yields in bioreactor cultivations, strain MG14 will also surpass reported yields of an IPTG-inducible promoter with *Y*_P/X_ of 0.92 and *q* of 0.025 g/(g h) (calculated based on data given in Jiao et al. (2017)), being additionally advantageous due to the proposed cheap, foam-reduced and self-inducible system.

To conclude, a self-inducible *B. subtilis* strain for surfactin synthesis induced upon oxygen-limitation has been developed. First, two interesting promoters, P_*narG*_ and P_*nasD*_ that are involved in anaerobic nitrate respiration, were tested. P_*narG*_ has the advantage of being an exclusively anaerobically activated promoter, but surfactin titers and yields were shown to not be competitive to the reference strain *B. subtilis* JABs24. However, strain MG14 with P_*srfA*_::P_*nasD*_ surpassed the titer of strain JABs24 by 200 mg/L at low oxygen availability and reached an exceptionally high *Y*_P/X_ of 1.007 g/g. Strain *B. subtilis* MG14 poses an interesting candidate for further surfactin production process development. Additional process developments are crucial to elaborate the high potential of strain *B. subtilis* MG14 as self-inducible surfactin producer in reduced foaming or even foam-free environments.

## Supplementary Information

**Additional file 1. **Information on plasmids and oligonucleotides used, main process data for bioreactor cultivations as well as the availability of nitrogen sources and their impact on target promoter expression during bioprocesses. **Figure S1**: Time course of CDW and P_*nasD*_expression under different availability of nitrogen sources.**Figure S2**: Time course of P_*narG*_ and P_*nasD*_ expression in MG12 and MG14 under different relative filling volumes. **Figure S3**: Availability of nitrate, nitrite and ammonium during cultivation of MG12, MG14 and JABs24 under different relative filling volumes. **Figure S4**: Strategies for oxygen-mediated bioreactor switch-processes using JABs24 and MG1.

## Data Availability

All discussed data have been included into the manuscript or in the additional file. Please turn to the corresponding author for all other requests.
