# Supplementary material for: Evaluation of an oxygen‐dependent self‐inducible surfactin synthesis in B. subtilis by substitution of native promoter PsrfA by anaerobically active PnarG and PnasD
Source: AMB Express. 2021 Apr 19;11:57. doi: 10.1186/s13568-021-01218-4 (PMC8055807; doi:10.1186/s13568-021-01218-4)
Supplement: Supplementary file 1 — Additional file 1. Information on plasmids and oligonucleotides used, main process data for bioreactor cultivations as well as the availability of nitrogen sources and their impact on target promoter expression during bioprocesses. Figure S1: Time course of CDW and PnasDexpression under different availability of nitrogen sources.Figure S2: Time course of PnarG and PnasD expression in MG12 and MG14 under different relative filling volumes. Figure S3: Availability of nitrate, nitrite and ammonium during cultivation of MG12, MG14 and JABs24 under different relative filling volumes. Figure S4: Strategies for oxygen-mediated bioreactor switch-processes using JABs24 and MG1. [file 13568_2021_1218_MOESM1_ESM.docx]

**Supplemental Material**

**Evaluation of an oxygen-dependent self-inducible surfactin synthesis in *B. subtilis* by substitution of native promoter P*_srfA_* by anaerobically active P*_narG_* and P*_nasD_***

Mareen Hoffmann, Alina Braig, Diana Stephanie Fernandez Cano Luna, Katharina Rief, Philipp Becker, Chantal Treinen, Peter Klausmann, Kambiz Morabbi Heravi, Marius Henkel, Lars Lilge^*^_,_ Rudolf Hausmann

Institute of Food Science and Biotechnology (150), Department of Bioprocess Engineering (150k), University of Hohenheim, Fruwirthstr. 12, 70599 Stuttgart, Germany

*****Corresponding author: Lars Lilge

Address: Fruwirthstraße 12, 70599 Stuttgart, Germany

Phone: +49-711-459-24728

Fax: +49-711-459-24722

Email: lars.lilge@uni-hohenheim.de

**Table S1** Plasmids used in this study

| Plasmid | Properties or insert | Reference |
| --- | --- | --- |
| pJOE4786.1 | cloning vector with *amp*^R^ | Jeske and Altenbuchner (2010) |
| pKAM0176 | *ori*_pUC18_, *bla*, P*_srfA_*  pJOE4786.1 (SmaI) + PCR product s1643-s1644 | This study |
| pKAM312 | *ori*_pUC18_*, bla*, *rop*, *ermC*, *amyE*′-[*ter*-P*_glcR_*-*lacZ*, *spcR*]- ′*amyE* | Morabbi Heravi and Altenbuchner (2018) |
| pKAM446 | *ori*_pUC18_*, bla*, *rop*, *ermC*, *amyE*′-[*ter*-P*_srfAA_*-*lacZ*, *spcR*]- ′*amyE*  Cut pKAM312 (AgeI-NdeI) Insert pKAM0176 (AgeI-NdeI) | This study |
| pJOE6743.1 | cloning vector with *amp*^R^, *spc*^R^, and *manP* for counter-selection | Wenzel and Altenbuchner (2015) |
| pRIK2 | pJOE4786.1 incorporating P*_narG_*-*srfA*; *amp*^R^ | This study |
| pRIK4 | pJOE6743.1 incorporating P*_narG_*-*srfA*; *amp*^R^, *spc*^R^, *manP* | This study |
| pKAM452 | *ori*_pBR322_, *rop*, *ermC*, *bla*, *amyE*′-[*ter*-P*_narG_*-*lacZ*-*spcR*]-′*amyE* | Hoffmann et al. (2020) |
| pPB1 | pJOE4786.1 incorporating P*_nasD_*-*srfA*; *amp*^R^ | This study |
| pPB2 | pJOE6743.1 incorporating P*_nasD_*-*srfA*; *amp*^R^, *spc*^R^, *manP* | This study |
| pSHX2 | *ori*_pBR322_, *rop*, *ermC*, *bla*, *amyE*′-[*ter*-P*_nasD_*-*lacZ*-*spcR*]-′*amyE* | Hoffmann et al. (2020) |

**Table S2** Oligonucleotides used in the current study

| Name | Sequence (5’ → 3’) | Application |
| --- | --- | --- |
| **Reporter strain P*_srfA_*-*lacZ*** | | |
| s1643 | **ATTATTAAGCTAGC**AACGCAGCAGTTTGGTTTAAAA | Amplification of P*_srfA_* |
| s1644 | **ATTATTAACATATG**ATTGTCATACCTCCCCTAATCTTT |  |
| **Promoter exchange P*_narG_*** | | |
| s1276^†^ | GCCCCTAAAACGGTGATGATG | Amplification of upstream flank of the *srfA* promoter |
| s1391 | C**GGTCTC**ACAGAAAAATAAAAATTTTTAAACCAAACTGC |  |
| s1392^†^ | **CGGTCTCC**ATGGAAATAACTTTTTACCCTTTAACG | Amplification of downstream flank of the *srfA* promoter |
| s1281 | GATTAGGAGATTATACGGATACTTTTGGTG |  |
| s1398^†^ | **GGGTCTCCTCTG**GCCGGTTTTTTTTGATCTTTG | Amplification of P*_narG_* for promoter exchange in *srfA* |
| s1399 | **CGGTCTCCC**CATGAGAGTTCACTCCTTCCGAGTC |  |
| **Promoter exchange P*_nasD_*** | | |
| s1276^†^ | GCCCCTAAAACGGTGATGATG | Amplification of upstream flank of *srfA* promoter |
| s1565 | **TGATAATTTGCTGCGGACG**TTACAACGATTCTTTCATGACATTTTTGTC |  |
| s1563^†^ | CGTCCGCAGCAAATTATCAG | Amplification of P*_nasD_* for promoter exchange |
| s1564 | CAGATGATCCGCTCCTTATCAAATG |  |
| s1566 | **GATAAGGAGCGGATCATCTG**ATGGAAATAACTTTTTACCCTTTAACGG | Amplification of downstream flank of *srfA* promoter |
| s1281 | GATTAGGAGATTATACGGATACTTTTGGTG |  |
| s1595 | GTATGTTCTGGATGCGTTAAC | Sequencing of P*_srfA_* promoter exchange |
| **Others** | | |
| s1637 | GCGTAATAGACTTTCAGGCGT | Confirmation of promoter-*lacZ* integration into *amyE* |
| s1638 | GCTTCATCCACCACATACAGG |  |
| s1639 | AGCCGCTGAAGAATATGG | Sequencing of promoter-*lacZ* integration into *amyE* |
| s1640 | CGTAATGGGATAGGTCAC |  |

^†^additionally used for sequencing

Added overhangs in bolt. Restriction sites highlighted by underlining.

**Table S3:** Summary of main process data, calculated Δ*t*_max_ yields and specific growth rates of *B. subtilis* bioreactor cultivations

| **Strategy** | **Reference pO_2_ set-point 20%** | **Fed-batch, constant aeration of 2 L/min** | | **Fed-batch, constant aeration of 1.2 L/min** | | **Fed-batch, constant aeration of 1.2 L/min** | |
| --- | --- | --- | --- | --- | --- | --- | --- |
| evaluation until | end of batch | after batch glucose consumption | end of process | after batch glucose consumption | end of process | after batch glucose consumption | end of process |
| *B. subtilis* strain | JABs24 | MG1 | | KM1016 | | MG14 | |
| Time of cultivation [h] | 24 | 22 | 40 | 26 | 46 | 19 | 46 |
| CDW_max_ [g] | 77.51 | 104.55 | 196.97 | 61.54 ± 9.21 | 147.52 ± 8.22 | 55.78 ± 13.24 | 150.18 ± 3.33 |
| Surfactin_max_ [g] | 57.43 | 51.65 | 104.57 | 48.47 ± 11.81 | 82.14 ± 0.91 | 15.49 ± 6.40 | 31.56 ± 3.74 |
| *Y*_P/X_ [g/g] | 0.885 | 0.718 | 0.718 | 0.902 ± 0.063 | 0.902 ± 0.063^†^ | 0.262 ± 0.062 | 0.262 ± 0.062^†^ |
| *Y*_X/S_ [g/g] | 0.133 | 0.845 | 0.845 | 0.466 ± 0.295 | 0.576 ± 0.186 | 0.293 ± 0.082 | 0.318 ± 0.057 |
| *Y*_P/S_ [g/g] | 0.161 | 1.381 | 1.381 | 0.313 ± 0.141 | 0.313 ± 0.141^†^ | 0.052 ± 0.015 | 0.052 ± 0.015^†^ |
| *q* [g/(g∙h)] | 0.147 | 0.308 | 0.308^†^ | 0.162 ± 0.011 | 0.162 ± 0.011^†^ | 0.059 ± 0.022 | 0.059 ± 0.022^†^ |
| *µ* [1/h] | 0.367 | 0.387 | 0.387^†^ | 0.576 ± 0.022 | 0.576 ± 0.022^†^ | 0.645 ± 0.001 | 0.645 ± 0.001^†^ |

^†^In these cases, the Δ*t*_max_ values were recorded in the batch phase and are hence the same when considering the complete process.

| 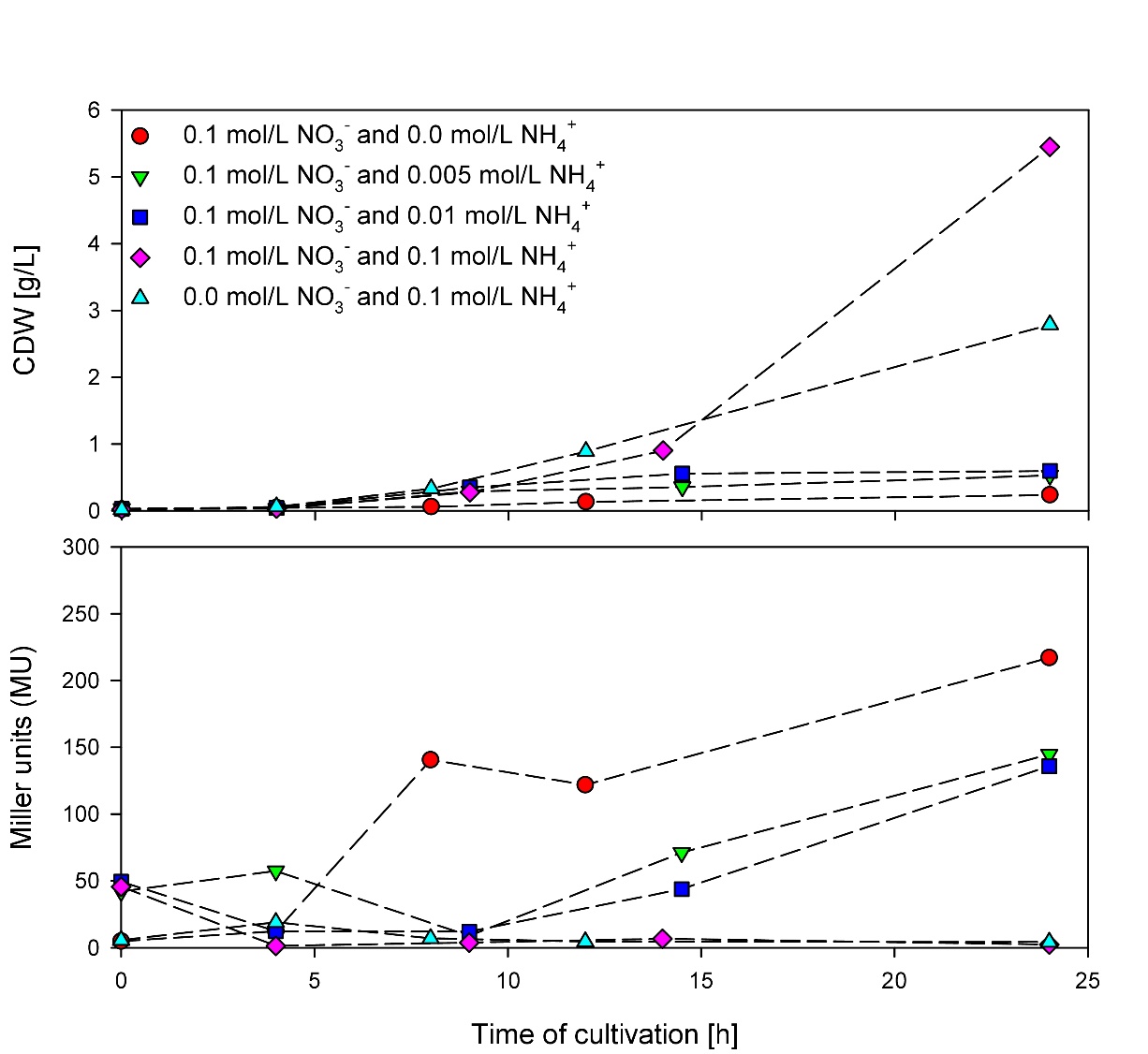 |
| --- |
| **Figure S1:** Time course of cell dry weight (CDW) [g/L] of strain *B. subtilis* MG5 (P*_nasD_*-*lacZ*) and corresponding Miller units (MU) determined by the Miller Assay representing expression of promoter P*_nasD_*. Data recorded in shake flask cultivations filled with 10% medium containing 40 g/L glucose and varying ammonium (NH_4_^+^) and nitrate (NO_3_^-^) concentrations (both [mol/L]). |

| 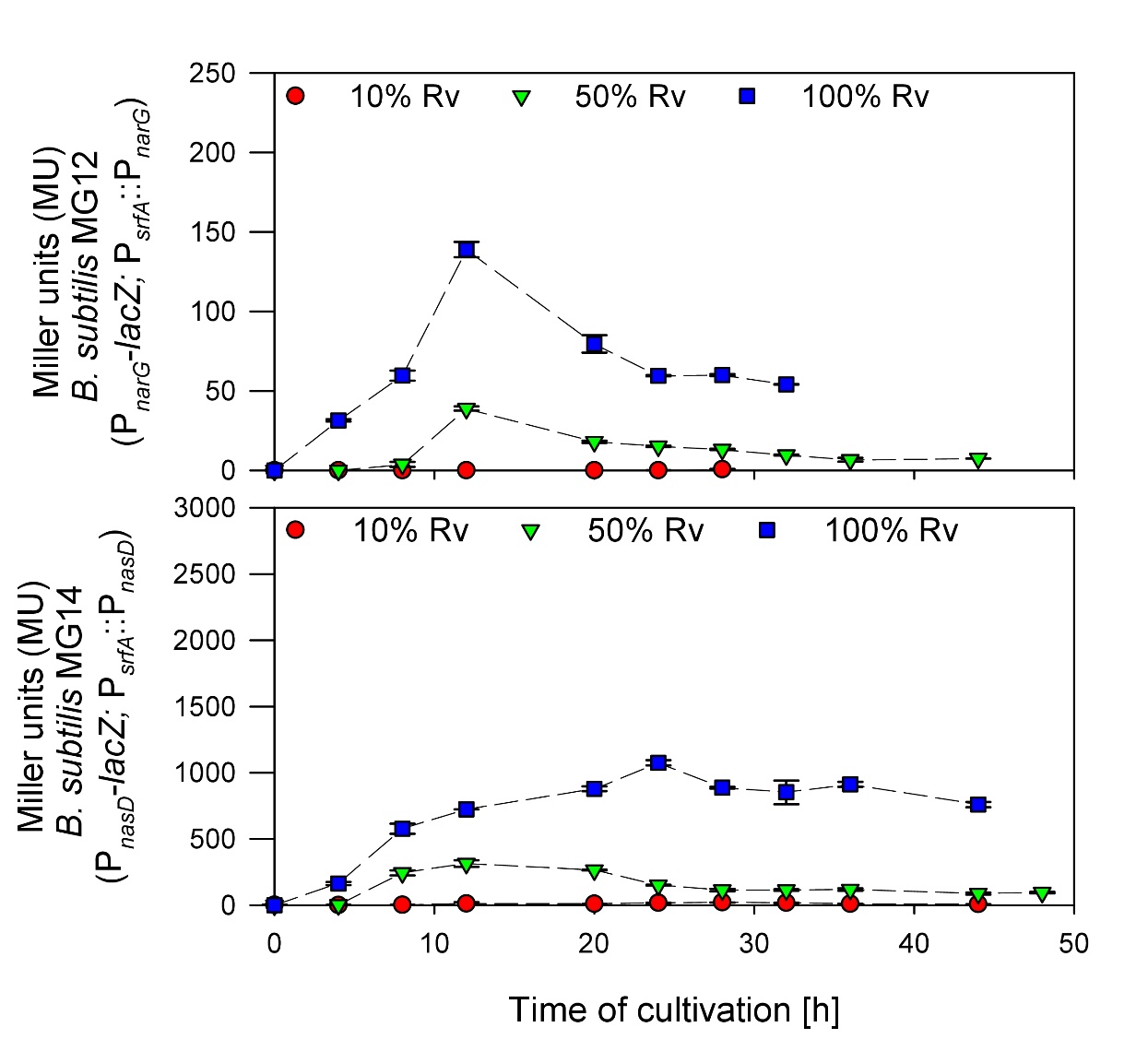 |
| --- |
| **Figure S2:** Time course of promoter expression P*_narG_* and P*_nasD_* represented by Miller units recorded until maximum cell dry weight (CDW_max_) of cultivation of strains *B. subtilis* MG12 (P*_narG_*-*lacZ*; *amyE*::[P*_narG_*-*lacZ*, *spcR*]) and MG14 (P*_srfA_*::P*_nasD_*; *amyE*::[P*_nasD_*-*lacZ, spcR*]) in a mineral salt medium containing 40 g/L glucose, 0.1 mol/L NH_4_^+^ and 0.1 mol/L NO_3_^-^ employing three different relative filling volumes of 10%, 50% and 100%. |

| a | 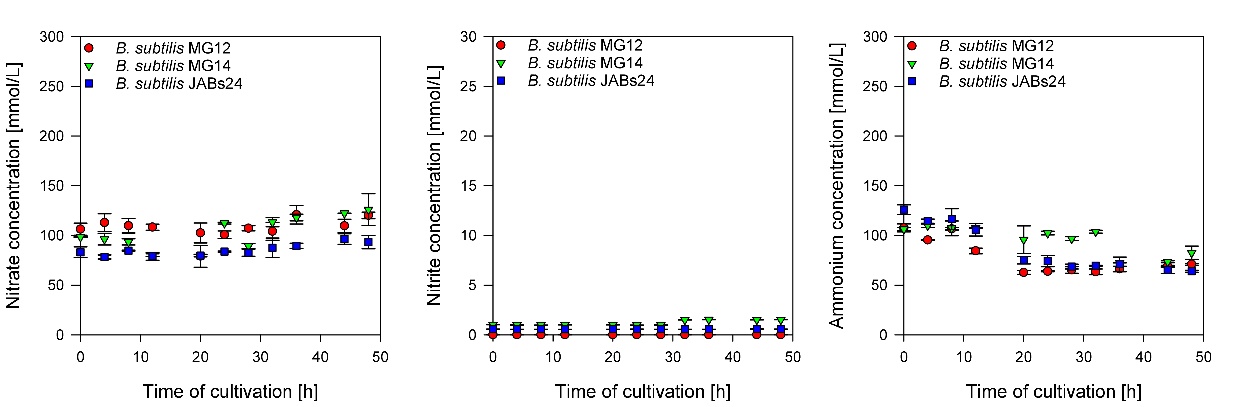 |
| --- | --- |
| b | 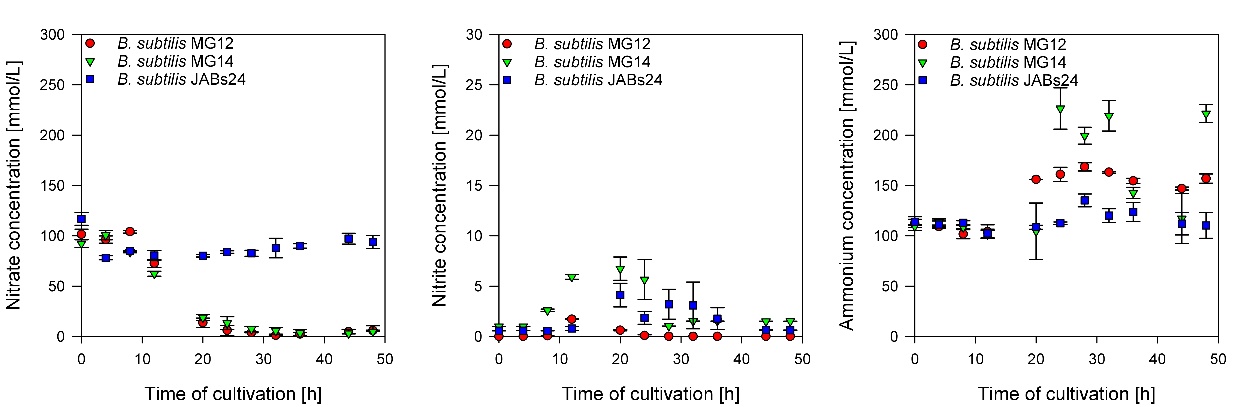 |
| c | 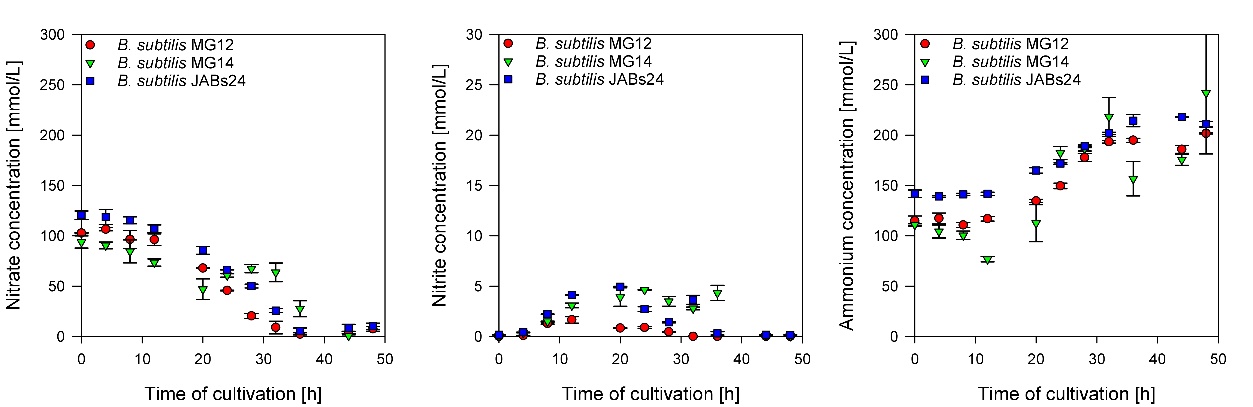 |
| **Figure S3**: Nitrate (left), nitrite (middle) and ammonium (right) concentrations (all [mmol/L]) recorded during the time course of cultivation of strains *B. subtilis* MG12 (P*_narG_*-*lacZ*; *amyE*::[P*_narG_*-*lacZ*, *spcR*]), MG14 (P*_srfA_*::P*_nasD_*; *amyE*::[P*_nasD_*-*lacZ, spcR*]) and JABs24 (reference) in a mineral salt medium containing 40 g/L glucose, 0.1 mol/L NH_4_^+^ and 0.1 mol/L NO_3_^-^ employing three different relative filling volumes of 10% (a), 50% (b) and 100% (c). | |

| a  b  c  d | 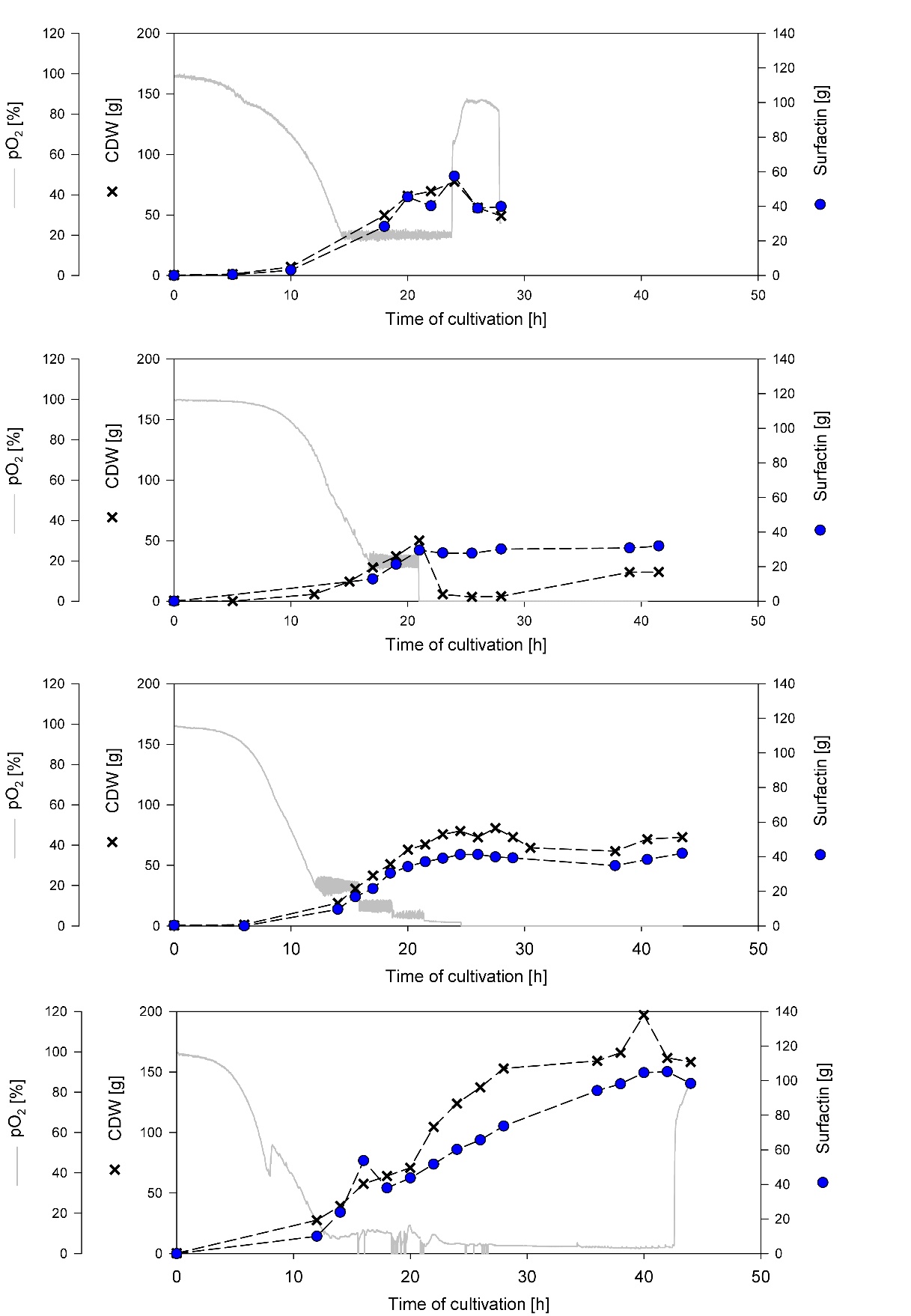 |
| --- | --- |
| **Figure S4:** CDW [g], surfactin [g] and pO_2_ [%] during the time course of cultivation of reference strains *B. subtilis* JABs24 (a and b) or MG1 (P*_narG_*-*lacZ*) (c and d) in bioreactors. The initial medium contained 20 g/kg glucose, 0.1 mol/kg NH_4_^+^ and 0.1 mol/kg NO_3_^-^. (a) Reference process with a pO_2_ set-point of 20% performed as batch. (b), (c) and (d) performed as fed-batch processes with different pO_2_ profiles (grey line). | |
